# The Kinetics of Anti–SARS-CoV-2 Antibodies in Pediatric Patients and the Characterization of Post–COVID-19 Condition at 6 Months After Infection: Protocol for a Longitudinal Observational Study

**DOI:** 10.2196/43344

**Published:** 2023-06-06

**Authors:** Eggi Arguni, Fatia Murni Chamida, Ratni Indrawanti, Desy Rusmawatiningtyas, Yunika Puspa Dewi, Ida Safitri Laksanawati

**Affiliations:** 1 Department of Child Health Faculty of Medicine, Public Health and Nursing Universitas Gadjah Mada Yogyakarta Indonesia; 2 Department of Integrated Laboratory Dr Sardjito General Hospital Yogyakarta Indonesia

**Keywords:** kinetics, anti–SARS-CoV-2 antibodies, post–COVID-19 condition, long COVID, pediatric patient, antibodies, COVID-19

## Abstract

**Background:**

Data regarding the kinetics of anti–SARS-CoV-2 antibodies and information about post–COVID-19 condition (colloquially known as “long COVID”) in children are scarce, especially in low-income countries. Even though cases of COVID-19 in children are less prevalent than adults, post–COVID-19 condition cases in children are high and have a burden that may impact their growth and development. There are other features of antibody kinetics in connection with SARS-CoV-2 infection that are yet unknown as of this writing, especially in children following infection. Furthermore, the long-term results, risk factors, and underlying pathophysiology are still uncertain. To better understand post–COVID-19 condition in children, it is necessary to further investigate the impact of clinically significant factors such multisystem inflammatory syndrome and disease severity among hospitalized survivors through their SARS-CoV-2 antibody response.

**Objective:**

We aim to analyze anti–receptor-binding domain SARS-CoV-2 immunoglobulin G antibodies over time and characterize the signs and symptoms of post–COVID-19 condition in pediatric patients at the time of diagnosis and at 2 weeks and 1, 3, and 6 months following infection.

**Methods:**

This is a longitudinal observational study in Indonesia. Pediatric patients diagnosed with COVID-19 by positive molecular assay using nasopharyngeal swab will be tested for anti–SARS-CoV-2 antibodies using the Roche Elecsys Anti-SARS-CoV-2 S assay at the time of diagnosis and at 2 weeks and 1, 3, and 6 months following infection. Antibody titer data will be reported as means and SDs. The respondents’ signs and symptoms will be observed up to 6 months after the onset of infection, including the vaccination event, reinfection, rehospitalization, and mortality. The clinical features will be reported as frequencies and percentages.

**Results:**

Participant enrollment began in February 2022. As of September 30, 2022, a total of 58 patients were enrolled. After data collection, results are expected to be analyzed in August 2023.

**Conclusions:**

This study will allow us to know the kinetics of anti–receptor-binding domain SARS-CoV-2 immunoglobulin G antibodies and data regarding post–COVID-19 condition up to 6 months following infection in the Indonesian pediatric population. Furthermore, this study has the potential to serve as a foundation for government decisions about vaccination programs and prevention measures.

**International Registered Report Identifier (IRRID):**

DERR1-10.2196/43344

## Introduction

COVID-19 is a pneumonia caused by the novel SARS-CoV-2 virus. It was first identified in Wuhan, China, in December 2019; it then spread rapidly across the globe and became an international public health emergency [1,2]. In March 2020, the World Health Organization (WHO) declared COVID-19 a pandemic [2]. SARS-CoV-2 is transmitted directly (droplet or person-to-person transmission through coughing, sneezing, or talking) and indirectly (contaminated objects and airborne) [1].

Data compiled by the WHO in 2021 showed that cases in children younger than 5 years from December 30, 2019, to September 13, 2021, were 1.8% of the world’s cases [3]. Meanwhile, cases of COVID-19 at the age of 5-14 years were 6.3% of the world’s cases. Older children (15-24 years) had more cases, which accounted for 14.5% of the world’s total cases. The American Academy of Pediatrics in 2022 stated that the total number of cases in children were 18.5% of the total COVID-19 cases in the world [4]. In Indonesia, there were 77,254 children who were positive for COVID-19 until December 21, 2020, accounting for 11.5% of all positive cases in Indonesia [5].

To date, there are many aspects that remain unknown about the antibody kinetics related to SARS-CoV-2 infection, especially in children. Understanding the kinetics of humoral response in the pediatric population could help to develop more effective vaccination programs in children [6,7].

The spike protein of SARS-CoV-2 contains a receptor-binding domain (RBD) that mediates the entry into human cells by binding to the angiotensin-converting enzyme 2 (ACE-2) receptor [8]. A positive correlation between anti-RBD immunoglobulin G (IgG) antibody levels and neutralizing antibodies has been described [9]. However, data on children are still inconsistent. Some studies described a persistence in neutralizing antibodies over a period of up to 8 months and reported that younger children have a higher titer compared to older siblings or adults [10,11]. In contrast, another study described higher anti–spike protein IgG antibody levels at younger ages, without high levels of neutralizing activity [12].

Around 15% to 24% of children with COVID-19 are asymptomatic. The symptoms of COVID-19 that most often appear in children are fever and cough [13]. Some respiratory symptoms that rarely appear in children include nasal congestion, sore throat, and dyspnea [14]. Other symptoms, such as headache, fatigue or myalgia, diarrhea, and vomiting, only occur in less than 10% to 20% of children [13].

Generally, the symptoms appear for approximately 6 days in children aged 5-17 years. Some of these symptoms can appear longer, whereas 4.4% of symptoms persist up to 4 weeks and 1.8% of symptoms are still present up to 8 weeks [15]. “Long COVID,” “post-COVID syndrome,” or “post-acute sequel of SARS-CoV-2 (PASC)” are terms that are often used to describe symptoms that persist around 4-12 weeks after being infected [16]. Other literature defines post–COVID-19 condition as new symptoms that appear and persist about 8 weeks after being infected with SARS-CoV-2 [17]. The prevalence of post–COVID-19 condition in children is different in each study, ranging from 4% to 66% [18]. The most common symptoms of post–COVID-19 condition are cognitive difficulties, headache, fatigue, fever, myalgia, cough, dyspnea, abdominal pain, diarrhea, and anosmia or altered sense of smell [16]. Different symptoms and severity are associated with different immune responses. Many aspects of the immune response in children are still unclear due to limited research in this population [12]. Accordingly, in this study, we aim to examine anti-RBD IgG antibodies over time and characterize the signs and symptoms of post–COVID-19 condition in pediatric patients at the time of diagnosis and at 2 weeks and 1, 3, and 6 months following infection.

## Methods

### Study Design

This study is a single-center, longitudinal observational study. Pediatric patients diagnosed with COVID-19 confirmed by molecular assay using nasopharyngeal swab will be recruited. The patients will be tested at the time of diagnosis and at 2 weeks and 1, 3, and 6 months after diagnosis to observe the kinetics of anti–SARS-CoV-2 antibodies. The Roche Elecsys Anti-SARS-CoV-2 S assay will be used to quantify the immunoglobulin M (IgM)/IgG–spike protein–RBD titer. We will also observe the signs and symptoms of the patients up to 6 months after infection to identify the sequelae of the post–COVID-19 condition cases.

### Study Setting

The study is being conducted in Dr Sardjito General Hospital, Sleman District, Yogyakarta Province, Indonesia. This is a tertiary referral hospital for Yogyakarta, Southern Central Java, and Eastern Indonesia regions.

### Sample Size

Samples were obtained by the total sampling method. We included all pediatric patients who met the inclusion criteria from February to April 2022.

### Participants

**Figure 1:**
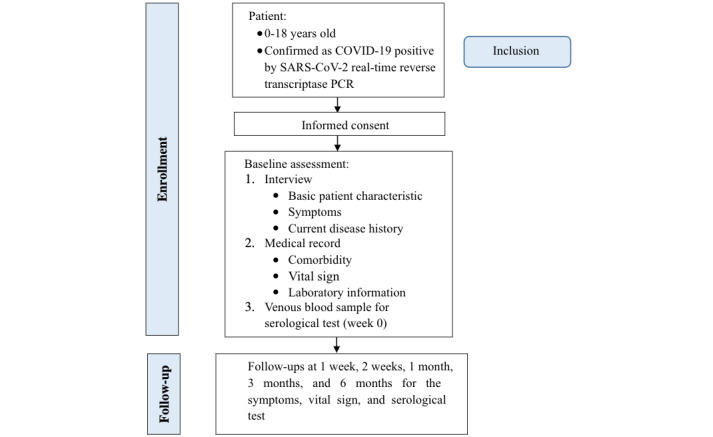
Study flowchart of inclusion criteria and data collection. PCR: polymerase chain reaction.

Patients will be observed prospectively after a confirmed diagnosis of COVID-19 in isolation wards at Dr Sardjito General Hospital. The inclusion criteria for this study are all patients aged 0-18 years who are confirmed as COVID-19 positive by SARS-CoV-2 real-time reverse transcriptase polymerase chain reaction (RT-PCR) with their legal guardian’s agreement to participate (Figure 1). There are no exclusion criteria for this study.

### Data Collection

Patient data will be retrieved from their medical records, which will include: (1) basic patient characteristic such as date of birth, sex, age, address, and referral case; (2) current disease history (symptoms, travel history, contact history in past 14 days, COVID-19 vaccination status, history of treatment in previous health facilities, and laboratory and imaging examination in previous health facilities); (3) comorbidity (diabetes; cancer; hypertension; obesity; heart disease; chronic obstructive pulmonary disease; chronic liver disease; chronic kidney disease; autoimmune disease; HIV; other infectious comorbidities such as hepatitis B and C, malaria, and dengue; and other immunodeficiencies such as long term steroid use and pelvic inflammatory disease); (4) vital signs and anthropometric parameters; (5) laboratory information (SARS-CoV-2 RT-PCR of the nasopharyngeal or oropharyngeal swab; complete blood count; electrolyte; blood glucose; ferritin; C-reactive protein; procalcitonin; lactate dehydrogenase; liver transaminases; serum blood urine nitrogen and creatinine; prothrombin time and activated partial thromboplastin time; D-dimer; lactate; blood gas analysis; cerebrospinal liquor examination; blood culture; broncho-alveolar liquid culture; and chest X-ray, computerized tomography scan, magnetic resonance imaging, or other imaging examination); and (6) clinical management data (drugs, intravenous fluid, supplements, oxygenation, blood transfusion, ventilator support, and other intensive care support).

Venous blood samples for serological tests will be drawn as a baseline when the diagnosis is made. Further serial blood samples will be collected at 2 weeks and 1, 3, and 6 months after the diagnosis (Figure 1). Samples will be collected in the clinical pathology laboratory. The data of any resolution or persistence of symptoms, such as fever, cough, runny nose, dyspnea, sore throat, seizure, cephalgia, malaise, nausea, vomitus, diarrhea, abdominal pain, myalgia, the loss of taste, the loss of smell, and dermatological disorder, in addition to patients’ vital signs, travel history, contact history, compliance with health protocols, any possible COVID-19 reinfection, COVID-19 vaccination status, therapy, and blood test result, will be recorded in each subsequent monitoring time point. A flowchart of the inclusion criteria and data collection of this study is shown in Figure 1.

### Blood Samples Collection and Storage

Samples will be collected in a vacutainer and sent to the pathology clinic laboratory for testing. The protocol is to proceed to centrifugation at 4000 rpm for 10 minutes. The samples will be uniquely coded and stored at –80 °C until use.

### The Roche Elecsys Anti-SARS-CoV-2 S Assay

The Roche Elecsys Anti-SARS-CoV-2 S assay is an immunoassay for the in vitro quantitative determination of total antibodies to the SARS‑CoV‑2 spike protein RBD in human serum and plasma. The test is intended as an aid to assess the adaptive humoral immune response, including neutralizing antibodies to the SARS‑CoV‑2 spike protein after natural infection with SARS‑CoV‑2 or in vaccine recipients. The assay is run on a Cobas e 411 analyzer, which utilizes an electrochemiluminescence immunoassay with the double-antigen sandwich principle. Calibration and quality control will always be performed before running the samples. The values obtained should fall within the defined limits. The analyzer automatically calculates the analyte concentration of each sample in U/mL with value ≥0.80 U/mL as a cut-off for positive anti–SARS-CoV-2 spike protein [19].

### Data Management Plan

All data are collected in an application-based data collection tool. Data will be stored on self-managed servers and only can be accessed by authorized research personnel.

### Data and Statistical Analyses

We will observe the kinetics of antibody response to the RBD of the spike protein of SARS-CoV-2 and the persistent signs and symptoms up to 6 months after the onset of infection.

Antibody titer data will be reported as means and SDs at baseline (when the diagnosis is made) and each monitoring time point (2 weeks and 1, 3, and 6 months after diagnosis).

The acute infection symptoms and post–COVID-19 follow-up clinical features will be reported in frequencies and percentages. The comparison of patient groups stratified by disease severity and RBD antibody will be analyzed and presented by graphs at the 1, 3, and 6-month follow-up time points.

### Ethics Approval

This study was reviewed and approved by the Medical and Health Research Ethics Committee, Faculty of Medicine, Public Health, and Nursing, Universitas Gadjah Mada (KE/FK/1288/EC/2021). The legal guardian of all participants will be provided with an information sheet detailing the aim and rationale for the research; inclusion and exclusion criteria; what will happen if they agree to take part; any risks of taking part in the study; the clarification of the process for ensuring anonymity and confidentiality, including any limits to confidentiality; what information will be held about them and who will have access to it; possible benefits; and plans for project report. All the legal guardians of the participants will then be required to sign the informed consent form before participating in the study. Additionally, all participants’ identifiable data will first be anonymized. The identifiable and anonymized data will be stored in separate secure cloud-based databases before being transferred to the Research Data Store. Finally, each participant will be compensated Rp 50.000,00 (US $3.37) at each monitoring time point.

## Results

Participant enrollment started in February and was extended to April 2022. The data collection was conducted up to 6 months following the last participant enrolled. As of September 30, 2022, a total of 58 patients were enrolled. After data collection, results are expected to be analyzed in August 2023.

## Discussion

### Expected Findings

This study aims to observe the kinetics of anti–SARS-CoV-2 antibodies and post–COVID-19 condition in children, up to 6 months after SARS-CoV-2 infection. SARS-CoV-2 consists of 4 main structural proteins, namely small envelope glycoprotein, membrane glycoprotein, nucleocapsid protein, and spike glycoprotein. The spike glycoprotein is divided into 2 subunits, S1 and S2. The S1 subunit includes RBDs that mediate viral binding to host cells via the ACE-2 receptor. Antibodies formed against the RBD on the S1 subunit have the potential to neutralize SARS-CoV-2 by preventing the binding of the virus’ ACE-2 receptor and endocytosis [20].

It takes 18 days from RT-PCR positivity to seropositivity (ie, antibody detection) [21]. Antibodies are detectable by the end of the first week in most patients, but progression can take weeks in patients with subclinical or mild infections. SARS-CoV-2 antibodies can be used to detect the presence of COVID-19 infection 1-3 weeks after the disease onset. In the acute phase, IgM peaks around 2-5 weeks following disease onset and then declines in 3-5 weeks before being undetectable. The immunoglobulin A anti-S1 antibodies decreased 1 month after diagnosis and remained detectable thereafter. The IgG peaks later, around 3-7 weeks following disease onset, and then persists for at least 6-8 weeks. Survivors with more severe symptoms show a stronger antibody response than asymptomatic and symptomatic survivors, in which IgM, IgG, and immunoglobulin A were found at higher titers and last longer in the body [22].

Post–COVID-19 condition is a condition where the symptoms of COVID-19 persist for a few weeks or months after SARS-CoV-2 infection [23]. Symptom usually persist around 4-12 weeks after being infected with COVID-19 [16,24]. The common symptoms are fever, fatigue, dyspnea, and myalgia [25]. Other symptoms that are present include chest pain, paresthesia, headache, hair loss, anosmia-ageusia or parosmia/anosmia, gastrointestinal symptoms, dizziness, weight loss of more than 5% of body weight, arthralgia, tremor, cough, palpitations, tiredness, difficulty concentrating, and increased need for sleep [25,26].

Older age and the presence of allergies are associated with higher risk of persistent symptoms after being infected with COVID-19. Those aged 6-18 years have a 3 times higher risk of persistent symptoms compared to children aged <2 years [27]. Children with allergies have a 2-3 times higher risk of persistent symptoms [27,28]. Patients with preexisting neurological comorbidities are also associated with persistent symptoms and have a 4 times higher risk of persistent symptoms after COVID-19 infection [28].

Research related to SARS-CoV-2 antibody kinetics in children is limited, and studies concerning the post–COVID-19 condition cases are scarce. It is important to conduct more research to determine children’s antibody response to SARS-CoV-2 infection and post–COVID-19 condition symptoms as the basis for patient management and forthcoming policy formulation.

This study will have recruitment and retention obstacles because it is difficult to get parental consent for serial blood sampling for children. This study will observe the development of post–COVID-19 condition symptoms and the kinetics of the COVID-19 antibodies for up to 6 months, making compliance from respondents and their families particularly important.

### Conclusions

At the completion of this study, we will be able to learn more about the dynamics of anti-RBD SARS-CoV-2 IgG antibodies and gain valuable information concerning post–COVID-19 condition in children in Indonesia for up to 6 months following infection. Additionally, this research may serve as a foundation for government decisions about vaccination programs and prevention measures.

## Abbreviations

ACE-2: angiotensin-converting enzyme 2
IgG: immunoglobulin G
IgM: immunoglobulin M
RBD: receptor-binding domain
RT-PCR: real-time reverse transcriptase polymerase chain reaction
WHO: World Health Organization

## Data Availability

The data sets generated during and/or analyzed during this study are available from the corresponding author on reasonable request to support scientific transparency.
